# Sleep-related breathing disorders beyond infancy following open spina bifida repair

**DOI:** 10.1007/s00431-026-07141-9

**Published:** 2026-06-06

**Authors:** Davin Lee, Danny Del Cid-Linares, Alexander Van Speybroeck, Kathryn A. Smith, Ramen H. Chmait, Thomas G. Keens, Sally L. Davidson Ward, Iris A. Perez

**Affiliations:** 1 Keck School of Medicine of University of Southern California, Los Angeles, CA USA; 2 Division of Pediatric Pulmonology and Sleep Medicine, Children’s Hospital Los Angeles, 4650 Sunset Blvd. Mail Stop 83, Los Angeles, CA 90027 USA; 3 Department of Pediatrics, Children’s Hospital Los Angeles, Los Angeles, CA USA; 4 Department of Obstetrics and Gynecology, Keck School of Medicine of University of Southern California, Los Angeles, CA USA

**Keywords:** Spina bifida, Myelomeningocele, Sleep-disordered breathing, Prenatal repair, Postnatal repair, Polysomnography

## Abstract

In patients with repaired open spina bifida, there are limited data on respiratory outcomes beyond the first year of life. We hypothesize that after 1 year of age there is 1) resolution of sleep-related breathing disorders (SRBD), 2) improvement of oxygenation and 3) no difference in SRBD between prenatally vs. postnatally repaired groups. We reviewed 67 patients with repaired open spina bifida and polysomnography (PSG) after age 1 year seen at Children’s Hospital Los Angeles between 2015–2025. Demographics, neural tube defect location, surgery type, PSG results, and respiratory support data were collected. There were 34/67 subjects with PSG before and after 1 year of age. Before 1 year of age, 19/34 had central sleep apnea (CSA), 33/34 had obstructive sleep apnea (OSA) and 30/34 required supplemental oxygen. At age 12 to 35 months, the prevalence of OSA (*p* < .001) and supplemental oxygen requirement (*p* = .006) decreased, however CSA persisted. There were no differences in SRBD between those with prenatal repair and postnatal repair of open spina bifida. There were 33/67 subjects with PSG after age 1 year only. In children ages 3 to 17 years, up to 92% had OSA, 11% had CSA, and 44% required supplemental oxygen.

*Conclusion*: In patients with repaired open spina bifida, OSA and supplemental oxygen requirement improved after 1 year of age, however, the majority still had SRBD without difference between those prenatally vs. postnatally repaired. Our findings highlight the importance of continued surveillance with PSG in this population.

**Table:** 

**What is Known:** • *Infants with prenatally or postnatally repaired open spina bifida have sleep-related breathing disorders* • *Current guidelines do not require formal evaluation with polysomnography as standard of care*	
**What is New:** • *After age 1 year, obstructive sleep apnea prevalence decreased, but persisted in the majority of patients* • *Oxygen supplementation requirement prevalence decreased*

**What is Known:**

• *Infants with prenatally or postnatally repaired open spina bifida have sleep-related breathing disorders*

• *Current guidelines do not require formal evaluation with polysomnography as standard of care*

**What is New:**

• *After age 1 year, obstructive sleep apnea prevalence decreased, but persisted in the majority of patients*

• *Oxygen supplementation requirement prevalence decreased*

## Introduction

Open spina bifida is a condition characterized by an open defect in the spinal canal due to incomplete closure of the neural tube during embryonic development, resulting in secondary changes in the brain including Chiari II malformation (CM-II) with hindbrain herniation [1]. Children with open spina bifida are at increased risk for developing sleep-related breathing disorders (SRBD) with previous studies suggesting a prevalence of up to 81% in children with myelomeningocele specifically [2, 3]. In the general pediatric population, SRBD may present with disrupted sleep architecture or poor gas exchange and have been associated with cognitive and cardiovascular problems if left untreated [4–6].

A growing number of studies have identified benefits of prenatal repair of open spina bifida [7]. Recently, Stark et al. found no significant difference in frequency or severity of SRBD in infants less than 1 year of age with prenatal repair compared to those with postnatal repair [8]. However, there are limited data on SRBD outcomes in children with prenatally or postnatally repaired open spina bifida as they age beyond 1 year. Thus, it is essential to understand the respiratory outcomes as these children grow older to guide decision-making for screening and management in the care of spina bifida patients.

The main goal of this study is to explore the prevalence and presentation of SRBD at least 1 year after surgical repair of open spina bifida. Additional goals include exploring the relationship between respiratory outcomes and potential factors that may predispose to chronicity or severity of SRBD. We hypothesize that after 1 year of age there is 1) resolution of SRBD, 2) improvement of oxygenation and 3) no difference in SRBD between prenatally vs. postnatally repaired groups.

## Methods

This study is a chart review of 67 patients with spina bifida between ages 1 and 21 years seen at CHLA between January 2015 and December 2025. Patients were included if they have undergone prenatal or postnatal open spina bifida repair and had a documented polysomnography (PSG) after the age of 1 year. Demographics, medical history, surgical history, maternal history, PSG results, and respiratory support data were collected. All polysomnograms were performed for clinical care of the patient and were reviewed by board-certified sleep medicine physicians in accordance with the American Academy of Sleep Medicine criteria at the time of the study.

Respiratory events were scored according to definitions from the American Academy of Sleep Medicine (versions 2.5, 2.6, 3.0) [9–11]. Obstructive apnea was defined as absence or decrease in air flow by ≥ 90% for at least 2 breaths associated with respiratory effort. Obstructive hypopnea was defined as decrease in flow by ≥ 30% with snoring, increased inspiratory flattening of the nasal pressure, or paradoxical breathing in association with ≥ 3% oxygen desaturation or arousal. Obstructive sleep apnea (OSA) was present if obstructive apnea–hypopnea index (OAHI) ≥ 1.5 events/h. Central apnea was defined as absence or decrease in flow by ≥ 90% for at least 20 s or breaths with absent respiratory effort and ≥ 3% oxygen desaturation, arousal, or heart rate < 50 beats per minute for 15 s. Central sleep apnea (CSA) was present if central apnea index (CAI) ≥ 5 events/h. Periodic breathing was defined as ≥ 3 central apneas with duration ≥ 3 s separated by ≤ 20 s of normal breathing. Severity of OSA was classified as mild (1.5–5 events/h), moderate (5–10 events/h), and severe (> 10 events/h) [12]. At our center, referring providers have the option to order a baseline, diagnostic polysomnogram in room air, or an oxygen titration with one of two protocols: 1. Begin oxygen supplementation for more than two desaturation episodes to an SpO2 of less than 89%, or 2. Begin oxygen supplementation for more than 5 min of sleep time with an SpO2 less than 89%.

Subjects were determined to be overweight if weight for length (WFL) or body mass index (BMI) was ≥ 85th percentile for their age. Subjects were obese if WFL or BMI was ≥ 95th percentile for their age.

Analysis was performed using Fisher’s exact, paired, and unpaired t-tests as appropriate to the type of data. This study was approved by the Institutional Review Board of Children’s Hospital Los Angeles.

## Results

We reviewed the data of 67 subjects. There were 36 (54%) males. There were 59 (88%) subjects with CM-II and 43 (64%) with ventriculoperitoneal (VP) shunt placement between 1 day of age and 19 months of age. Location of neural tube defects (NTD) were thoracic (2), thoracolumbar (1), lumbar (26), lumbosacral (18), and sacral (12). There were 8 subjects with unknown location of NTD. Of the 67 subjects, 47 underwent prenatal repair of the open spina bifida. There were 43 (64%) subjects with mothers who had early positive prenatal care and/or folic acid supplementation during pregnancy. Forty-two subjects only had a diagnosis of NTD, with the others having comorbid pulmonary (11), cardiac (2), seizure (7), gastroesophageal reflux (4) or both pulmonary and cardiac conditions (1). There was 1 subject with tracheostomy whose data was not included in analysis of OSA.

Cohorts were grouped by age at PSG with some subjects having PSGs across age groups. Subjects had on average 3.4 PSGs (range 1–10 PSGs per subject). Of total 67 subjects, 38 had PSG between ages 12 to 35 months and 34/38 had PSG before age 12 months. Fourteen of the 38 had repeat PSGs at age 3 to 7 years; of the 14, 1 had repeat PSG between 8 to 12 years and not repeated since.

**Fig. 1:**
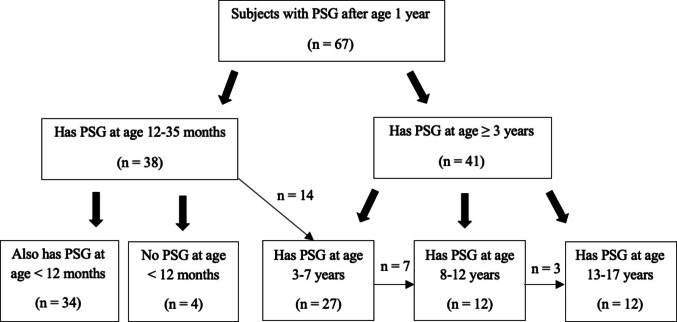
Cohorts were defined by age at PSG. Key cohorts were defined as subjects with PSG at 12 to 35 months, with and without PSG before age 12 months. Additional cohorts include subjects aged 3 to 7 years, 8 to 12 years, and 13 to 17 years at time of PSG

A total of 41 subjects had PSG at age 3 years or older with 27 subjects having PSG at age 3 to 7 years. Seven of the 27 had repeat PSG at age 8 to 12 years. Of the 12 with PSG at age 8 to 12 years, 3 also had PSG at age 13 to 17 years (Fig. 1).

### Patients with PSG before age 12 months and at 12–35 months

There were 34 subjects with PSG before and after the age of 1 year. Average postnatal age at first PSG was 7.7 ± 14 weeks. Average postconception age was 40.3 ± 4.7 weeks. Before the age of 1 year, 19/34 (56%) had CSA (average CAI 17.4 ± 13.4 events/h) and 33/34 (97%) had OSA (average OAHI 25.0 ± 22.1 events/h). There were 30/34 (88%) who required supplemental oxygen during the PSG. Of note, CAI was not differentiated between time periods with and without supplemental oxygen in these PSGs.

**Fig. 2:**
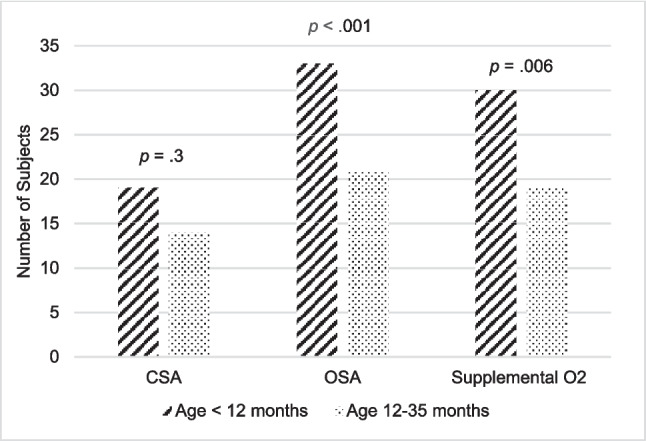
After 1 year of age, there was no significant difference in CSA prevalence, however the prevalence of OSA and supplemental oxygen requirement decreased (*n* = 34)

The average age at first PSG after the age of 1 year was 19.6 ± 4 months. At the age of 12 to 35 months, there was a decrease from 97 to 62% in the OSA prevalence from before age 12 months (*p* < 0.001). The OAHI also decreased from 25.0 ± 22.1 events/h before age 1 year to 3.9 ± 5.0 events/h (*p* < 0.001). The prevalence of supplemental oxygen requirement decreased from 88 to 56% (*p* = 0.006). Supplemental oxygen was initiated during the PSG for 17/19 (89%) subjects. There was no significant difference in CSA prevalence. There were 3 subjects who did not have CSA as infants who then had CSA after age 1 year. These subjects were started on oxygen supplementation during their first PSG as infants and the CAI prior to oxygen supplementation was not reported. The CAI of those who initially had CSA as infants was decreased (*p* = 0.003) (Fig. 2). The percent of sleep time with oxygen saturation (SpO_2_) below 90% decreased (*p* = 0.03), however there was no significant difference in SpO_2_ baseline or SpO_2_ nadir. There was no difference in baseline partial pressure of end-tidal CO_2_ (P_ET_CO_2_) or maximal P_ET_CO_2_ (Table 1).

**Table 1:** PSG results of a cohort before 12 months of age compared to results at 12 to 35 months of age (*n* = 34)

Result	0–12 Months	12–35 Months	*p*
Age	7.7 ± 14 weeks	19.6 ± 4 months	-
CSA (*n*)	19	14	0.3
CAI (*n* = 19)	17.4 ± 13.4 events/h	6.3 ± 4.1 events/h	**0.003**
OSA (*n*)	33	21	** < 0.001**
OAHI (*n* = 33)	25.0 ± 22.1 events/h	3.9 ± 5.0 events/h	** < 0.001**
SpO_2_ baseline	95 ± 4%	96 ± 1%	0.4
SpO_2_ nadir	78 ± 9%	79 ± 8%	0.6
Percent sleep time with SpO_2_ < 90%	2.5 ± 4%	1 ± 0.8%	**0.03**
Supplemental oxygen	30 subjects	19 subjects	**0.006**
P_ET_CO_2_ baseline	34 ± 5 mmHg	37 ± 5 mmHg	0.05
P_ET_CO_2_ maximum	47 ± 7 mmHg	50 ± 8 mmHg	0.2

Of the 34 subjects with PSG before and after the age of 1 year, 17 (50%) had prenatally repaired open spina bifida. Before 1 year of age, 13/17 (76%) had CSA, all had OSA, and 16/17 (94%) required supplemental oxygen. Of the 17 subjects with postnatally repaired open spina bifida, 7/17 (41%) had CSA, 16/17 (94%) had OSA, and 14/17 (82%) required supplemental oxygen. In the prenatally repaired group at age 12 to 35 months, 7/17 (41%) had CSA, 11/17 (65%) had OSA, and 7/17 (41%) required supplemental oxygen. In the postnatally repaired group, 7/17 (41%) had CSA, 10/17 (59%) had OSA, and 12/17 (71%) required supplemental oxygen. Between prenatally repaired and postnatally repaired groups, there was no significant difference in CSA (*p* = 0.08, *p* = 1) or OSA (*p* = 1, *p* = 1) before and after 1 year of age. There was also no difference in supplemental oxygen requirement (*p* = 0.6, *p* = 0.2).

Of 16 subjects who were overweight/obese, 8/16 (50%) had CSA and 10/16 (63%) had OSA. Of 18 subjects who were normal weight or underweight, 6/18 (33%) had CSA and 11/18 (61%) had OSA. There was no significant difference in prevalence of CSA (*p* = 0.5) or OSA (*p* = 1) between the groups.

### Patients with PSG at 1–2 years of age

There were 38/67 subjects who had PSG between 1 to 2 years of age. Of the 38 subjects, 34 had PSG before and after 1 year of age; the remaining 4 had PSG after 1 year of age only. The average age at PSG was 19.7 ± 4 months. There were 14/38 (37%) with CSA. OSA was present in 24/38 (63%) subjects. OSA severity was mild for 13/24 (54%) subjects, moderate for 6/24 (25%) subjects, and severe for 5/24 (21%) subjects. There were 23/38 (61%) who required supplemental oxygen. In this age group, 37/38 (97%) subjects had CM-II, of whom 14/37 (38%) had CSA and 23/37 (62%) had OSA. VP shunt was present in 21/38 (55%) subjects, of whom 6/21 (29%) had CSA and 16/21 (76%) had OSA.

### Patients with PSG ≥ 3 years of age

**Fig. 3:**
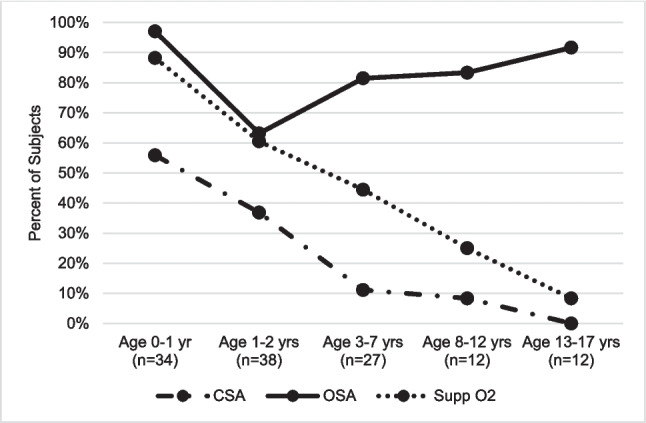
Prevalence of CSA and supplemental oxygen requirement downtrend in older age groups, while OSA prevalence downtrends until age 2 years

There were 27 subjects with PSG between ages 3 to 7 years. Average age at PSG was 3.7 ± 1 years. In this age group, 3/27 (11%) had CSA, 22/27 (81%) had OSA, and 12/27 (44%) required supplemental oxygen. There were 12 subjects with PSG between ages 8 to 12 years. Average age at PSG was 8.8 ± 1 years. In this age group, 1/12 (8%) had CSA, 10/12 (83%) had OSA, and 3/12 (25%) required supplemental oxygen. There were 12 subjects with PSG between ages 13 to 17 years. Average age at PSG was 14.5 ± 2 years. In this age group, 11/12 (92%) had OSA and 1/12 (8%) required supplemental oxygen. None had CSA. There was a downtrend in percentage of children with CSA and supplemental oxygen requirement with age. There was a downtrend in percentage of children with OSA until age 2 years (Fig. 3).

### Respiratory support and treatment outcomes

The use of supplemental oxygen was determined by the patients’ primary provider. There were 29/67 (43%) subjects with documented discontinuation of supplemental oxygen at average age 2.9 ± 2 years (range 1–8 years). There were 14/67 (21%) subjects who utilized positive airway pressure (PAP) support. PAP support was discontinued at average age 14 ± 5.3 years (range 5–21 years). Two subjects discontinued PAP (age 8 and 21 years) and transitioned to supplemental oxygen. There were 11 subjects who had adenotonsillectomy at average age 5.8 ± 5 years. PSG was obtained on average 11.5 months (range 1 to 35 months) after adenotonsillectomy; all subjects had OSA.

## Discussion

This study aimed to explore the prevalence of SRBD and need for respiratory support in patients with spina bifida beyond infancy. Our study showed that CSA prevalence was 56% in infancy and persisted at 41% by ages 12 to 35 months, however the severity of CSA improved. Despite persistence of CSA among the youngest age group, prevalence was as low as 0–11% among children ages 3 to 17 years. Prevalence of OSA significantly decreased from 97% in infancy to 62% by ages 12 to 35 months. Severity of OSA also improved, however most patients continued to have OSA. In older children, OSA prevalence was as high as 81–92%. We found higher prevalence of SRBD compared to previous studies where SRBD was described in 42–81% of patients with open spina bifida [2, 13, 14].

OSA is known to be associated with increased body weight and large tonsil status which may cause narrowing of the airway due to excess fatty or lymphoid tissue [15, 16]. We found no significant difference in SRBD prevalence between overweight/obese and healthy weight groups. Furthermore, there was presence of OSA following adenotonsillectomy. Our findings support previous studies where OSA persisted following adenotonsillectomy in children with myelomeningocele [14, 17]. Therefore, these patients require continued surveillance following adenotonsillectomy. The lack of association between OSA with weight or tonsil status suggests other contributory factors for OSA in this population.

Among our subjects with CM-II, we found that 38% had CSA and 62% had OSA, compared to previous studies which report SRBD prevalence of 35–50% among patients with CM-II [18, 19]. Luigetti et al. described a case of a child with open spina bifida and CM-II who had significant improvement of OSA following ventriculoatrial shunt placement, suggesting that elevated intracranial pressure may play a role in SRBD [20]. Previous studies have identified association of myelomeningocele with anatomic and functional abnormalities of the brainstem including compression, vascular insufficiency, injury or abnormal development to explain respiratory disorders in patients with open spina bifida [21, 22]. Other anatomical variations or differences in neuromuscular activity may be considered as potential contributing factors.

We found that the prevalence of supplemental oxygen requirement decreased from 88 to 56% after 1 year of age. For those where oxygen supplementation was discontinued, the average age of discontinuation was 2.9 years. There are children who required supplemental oxygen beyond the age of 3 years with prevalence of 44% among young children (ages 3 to 7 years) and 8% among teenagers. Supplemental oxygen is a therapeutic option for SRBD in infants and children who are not candidates for surgical intervention or positive airway pressure therapy. Prior studies have demonstrated that supplemental oxygen can reduce the frequency of central apneas, periodic breathing, and OSA [23–25]. Brockbank et al. reported that infants with a mean age of 13 weeks who were initiated on supplemental oxygen at 15 weeks exhibited a significant reduction in the apnea–hypopnea index (AHI) and improved oxygenation [23]. Similarly, Aljadeff et al. observed decreased hypopnea density, a lower obstructive apnea index, and reduced paradoxical breathing in children aged 2 to 8 years with OSA [24].

Infants demonstrate high loop gain—reflecting unstable ventilatory control—predisposing to upper airway collapse. In adults with obstructive sleep apnea and elevated loop gain, supplemental oxygen has been shown to reduce loop gain and decrease AHI [26]. This mechanism provides a rationale for the use of supplemental oxygen in infancy and childhood, particularly in patients with unstable ventilatory control. In our study, PAP support was found in 21% of subjects and was discontinued at average age of 14 years. These findings suggest that SRBD following repaired open spina bifida often require years of therapeutic intervention.

In our infant cohort, the majority have SRBD, which is consistent with the results of Stark et al. and Bendel-Stenzel et al. [8, 27]. Similarly, we found no difference in SRBD prevalence between prenatally and postnatally repaired groups as infants. Our study suggests there is no difference beyond 1 year of age, further supporting the idea that timing of open spina bifida repair does not affect prevalence of SRBD.

Limitations of this study include retrospective design, lack of standardized timing of PSG, and single center data collection. Potential confounding variables include additional surgeries, medications, comorbid conditions, and interim medical events. Additionally, potential confounding factors to development of SRBD such as CM-II severity and VP shunt revision data were not collected. Notably, there were 3 subjects who appear to have developed CSA after age 1 year. These subjects were started on oxygen supplementation during their first PSG as infants, with the oxygen therapy possibly obscuring the scoring of central apneas. Almost all subjects who were found to have supplemental oxygen requirement were initiated on oxygen supplementation during the PSG per institution protocol; therefore, our results may underestimate the true CAI and CSA prevalence. Complications related to CM-II, VP shunts, and epilepsy (for one subject), may also explain the later development of CSA in those patients. Furthermore, there were 38 patients who had PSG before 1 year of age only who were excluded from the study for various reasons, including lack of follow-up PSG, PSG ordered but not completed, transfer of care to a different institution, and patients lost to follow up. The exclusion of these subjects may have impacted the generalisability of the results.

Our study demonstrates that SRBD commonly persist beyond infancy among individuals with repaired open spina bifida. Many of these patients require intervention with supplemental oxygen or PAP for years beyond infancy. Our findings support the need for continued surveillance and development of screening guidelines with PSG to ensure early detection and treatment of SRBD in patients with spina bifida.

## Data Availability

No datasets were generated or analysed during the current study.
